# Multiple arterial aneurysms involving the thoracoabdominal aorta and renal arteries in a young patient with possible heritable etiology: Two-stage open surgical repair management

**DOI:** 10.1016/j.jvscit.2025.102084

**Published:** 2025-12-06

**Authors:** Annarita Santoro, Marian Broasca, Horatiu Moldovan, Fiorenza De Lisio, Francesca Sanvito, Germano Melissano

**Affiliations:** a Division of Vascular Surgery, IRCCS San Raffaele Scientific Institute, Vita-Salute San Raffaele University, Milan, Italy; b "Carol Davila" University of Medicine and Pharmacy Bucharest, Clinical Emergency Hospital Bucharest, Bucharest, Romania; c Pathology Unit, Department of Experimental Oncology, IRCCS Ospedale San Raffaele, Vita-Salute San Raffaele University, Milan, Italy

**Keywords:** Aneurysm, Multilevel arterial aneurysm, Renal artery aneurysm, Thoracic aorta, Thoracoabdominal aneurysm, Visceral aneurysm

## Abstract

Multilevel arterial aneurysm refers to aneurysms in multiple sites, such as the aorta, cerebral, or peripheral arteries, often caused by atherosclerosis, heritable thoracic aortic disease, inflammation, or infection. We report the case of a 48-year-old patient with multilevel arterial aneurysm managed with staged open surgical repair. The first surgery addressed an 11-cm abdominal aortic aneurysm and renal artery aneurysms, followed by thoracoabdominal aortic aneurysm repair. Both procedures were uneventful, with successful grafting and preserved organ perfusion. Six-month follow-up computed tomography scan showed no complications. Postoperative genetic testing revealed two variants of uncertain significance (PKD1, FBN1). Open surgical repair proved safe and effective in a high-volume center.

Multilevel arterial aneurysm (MAA) is an uncommon pathological entity characterized by the presence of aneurysms occurring in different arterial segments in a synchronous or metachronous fashion. This condition may result from underlying atherosclerosis, heritable thoracic aortic disease (HTAD), inflammatory disease, or infections.^1^^,^^2^

The occurrence of MAA has been increasingly reported in the literature. Early studies primarily focused on cases of synchronous infrarenal abdominal aortic aneurysms (AAAs) and peripheral artery aneurysms. However, MAAs have also been described in different arterial segments with peculiar challenges in their management.^3^

We present the case of a 48-years-old patient with a very unusual-looking MAA treated by means of two-stage open surgical repair (OSR).

## Case Report

**Fig 1:**
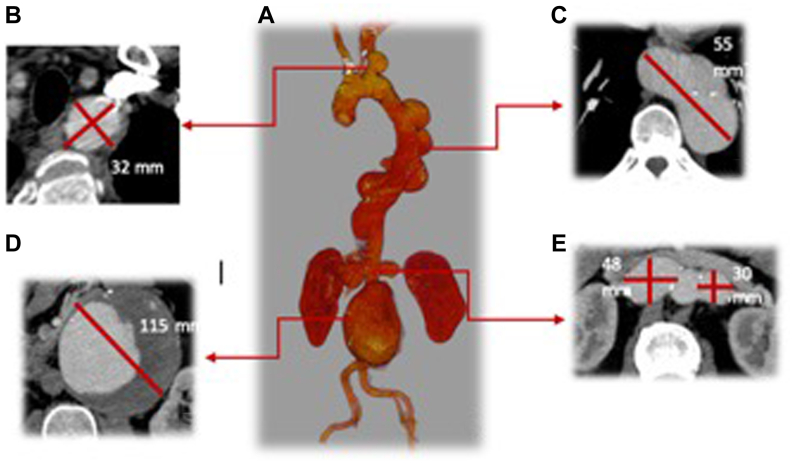
Preoperative computed tomography angiography three-dimensional volume rendering reconstruction **(A)**, showing a left subclavian artery aneurysm (maximum diameter of 32 mm) **(B)**, multiple descending thoracic aorta aneurysms (maximum diameter of 55 mm) **(C)**, a juxtarenal aortic aneurysm with a maximum diameter of 115 mm **(D)**, bilateral renal artery aneurysms (left renal artery maximum diameter of 48 mm, right renal artery maximum diameter of 30 mm) **(E)**.

The patient was referred from another European Institution for MAA. The subject is a practicing physician who was asymptomatic, except for a recent-onset and persistent cough. Computed tomography angiography showed a left subclavian artery aneurysm (32 mm), multiple descending thoracic aorta aneurysms (TAAAs) (55 mm), a juxtarenal AAA (115 mm), and bilateral renal artery aneurysms (left renal artery 48 mm, right renal artery 30 mm). No calcifications, thrombus, or other signs of degenerative atherosclerotic aneurysm were detected (Fig 1). His genetic panel at the time of the referral to our Institution was negative.

**Fig 2:**
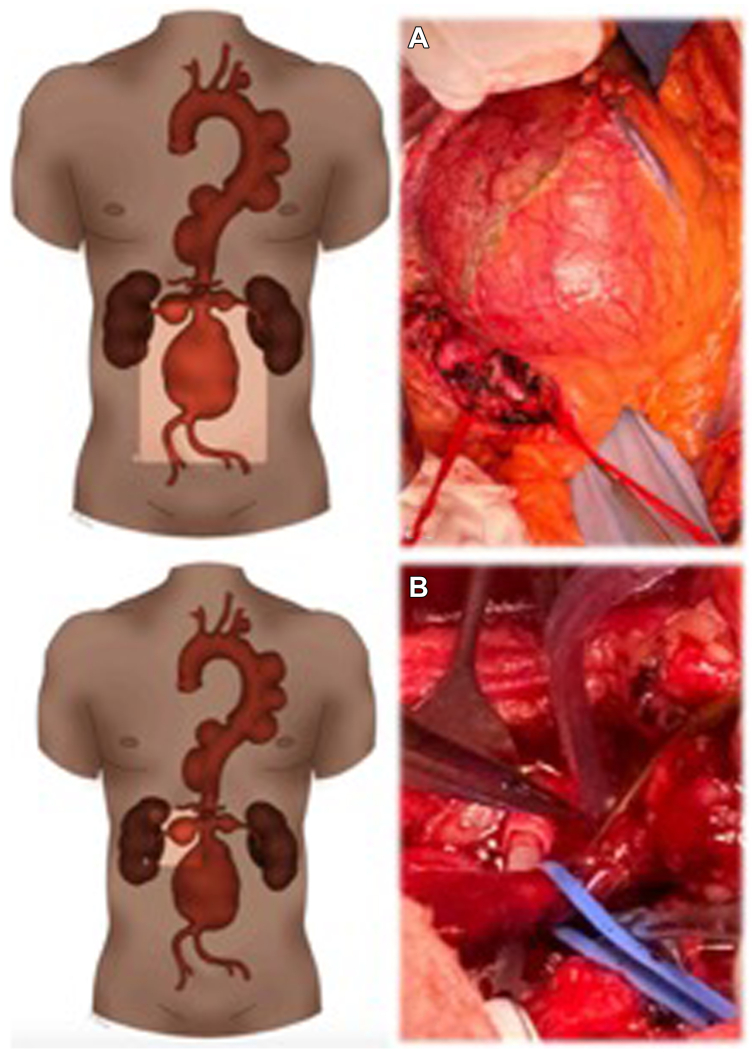
**(A)** First-stage open procedure, through a midline laparotomy and intraoperative finding of a large juxtarenal abdominal aortic aneurysm (AAA). **(B)** Right renal artery was approached and exposed through a Kocher maneuver by incising the retroperitoneum at the level of the right line of Toldt from the right colon, extending peripheral margins to the duodenum. Both renal arteries were cannulated with a 9F occlusion-perfusion catheter and perfused with 4 °C Custodiol solution (Dr Franz-Kohler Chemie GmbH, Bensheim, Germany).

**Supplementary Fig 1:**
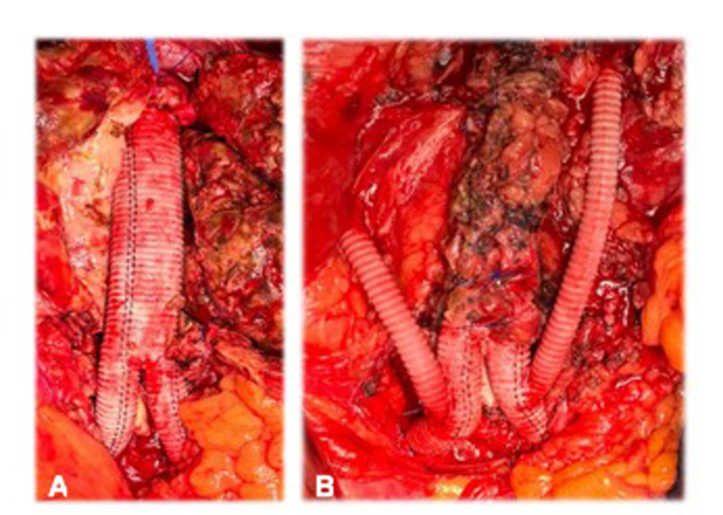
**(A and B)** Aortobisiliac reconstruction with a 20 × 10 mm Hemashield Platinum graft, and bilateral renal arteries ligature with bilateral retrograde bypass from the iliac limbs to the renal arteries, with a 6-mm Intergard Heparin graft.

A two-staged surgical repair was planned. The first procedure was an OSR of the AAA and renal arteries aneurysms, and the second step was a TAAA OSR. The first procedure was an aortobisiliac reconstruction with a 20 × 10 mm Hemashield Platinum and bilateral renal arteries ligature with bilateral retrograde bypass from the iliac limbs to the renals, with a 6-mm Intergard Heparin (Fig 2). While the left renal artery was approached and exposed through a standard retroperitoneum incision, for the right renal artery, a Kocher maneuver was performed by incising the retroperitoneum at the level of the right line of Toldt from the right colon, extending the peripheral margins to the duodenum. Both renal arteries were cannulated with a 9F occlusion-perfusion catheter and perfused with 4 °C Custodiol solution as previously described in patients undergoing open complex AAA OSR in our institution^4^ (Supplementary Fig 1, online only). The postoperative course was uneventful, and the patient was discharged on postoperative day 9.

One month after the first operation, the second planned TAAA procedure was performed according to our standard approach, as previously extensively described.^5^, ^6^, ^7^, ^8^, ^9^, ^10^

The surgical incision was performed in the fifth intercostal space. A cryoablation therapy of T3-T7 intercostal nerves was performed for postoperative pain management.^11^

**Fig 3:**
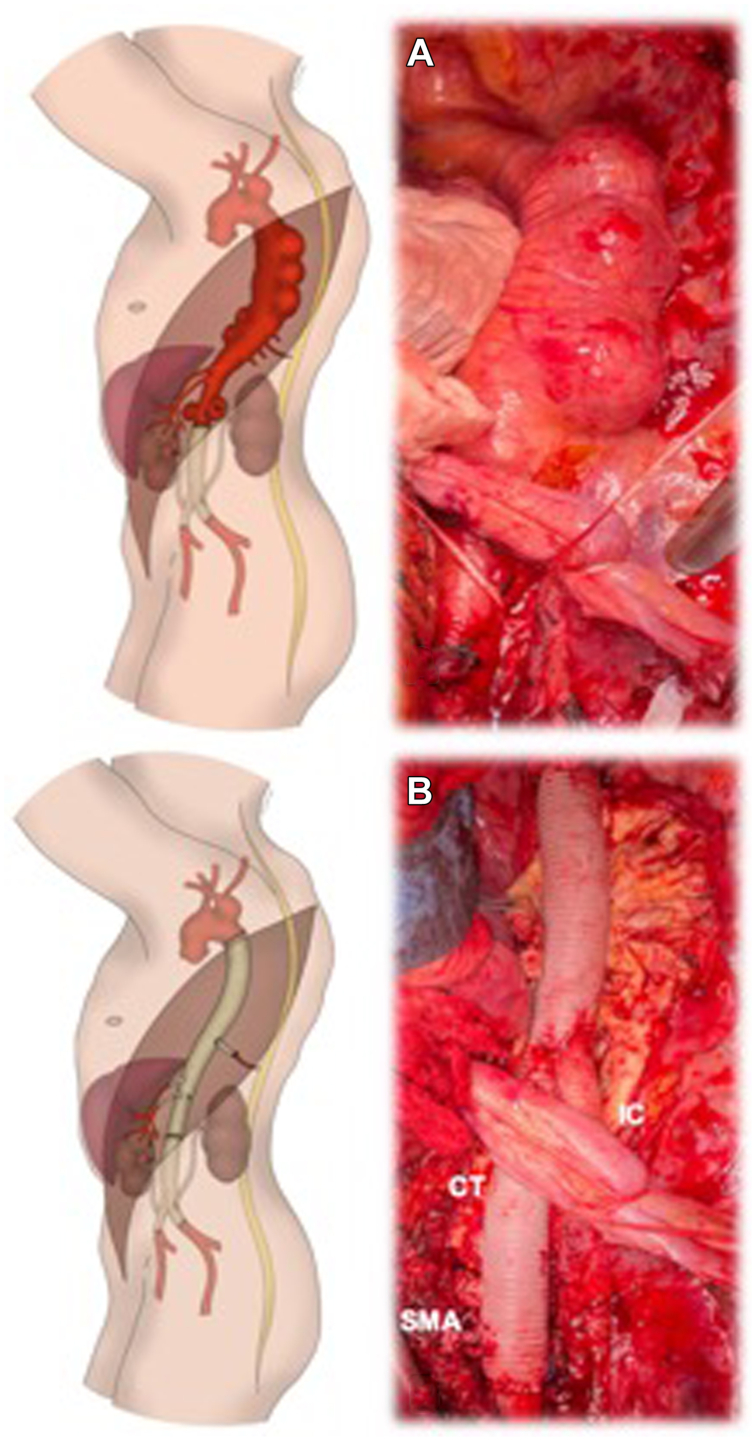
Intraoperative finding of multiple saccular aneurysms in the descending thoracic aorta **(A)** and aortic reconstruction with a 20-mm multibranched “Coselli” aortic graft, with ligation of the renal branches. **(B)** The celiac trunk and superior mesenteric artery (*SMA*) were anastomosed to the graft branches.

After exposure of the descending thoracic aorta and proximal neck and distal surgical graft control, left heart bypass was performed. A 20-mm multibranched Coselli aortic graft was used; the renal branches were ligated, the descending thoracic aorta and the abdominal aorta were replaced, and the celiac trunk and superior mesenteric artery were anastomosed to the graft branches (Fig 3). Two intercostal arteries were identified as suitable for reimplantation at the level of T8-T9, and were reimplanted with a 12-mm Hemashield from the main graft.

**Supplementary Fig 2:**
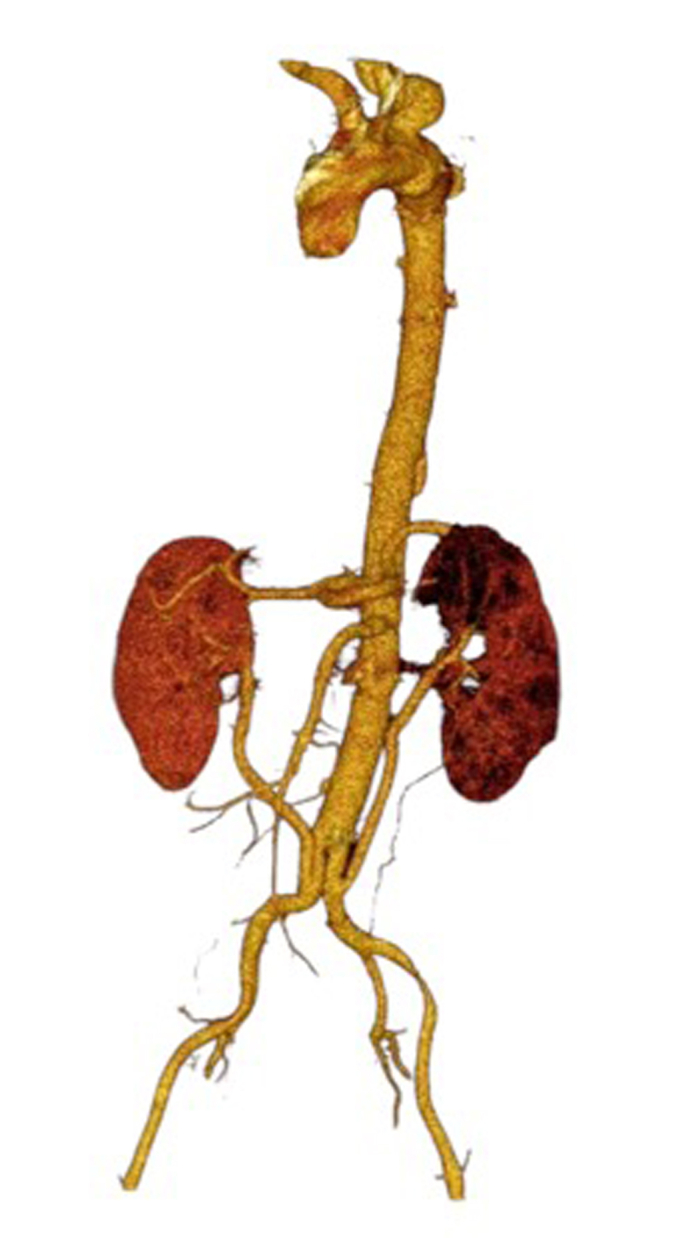
Postoperative computed tomography angiography three-dimensional volume rendering reconstruction showing good results in terms of thoracoabdominal reconstruction, retrograde iliac-renal artery bypasses bilaterally patent, patency of the visceral arteries, without any sign of complication.

**Supplementary Fig 3:**
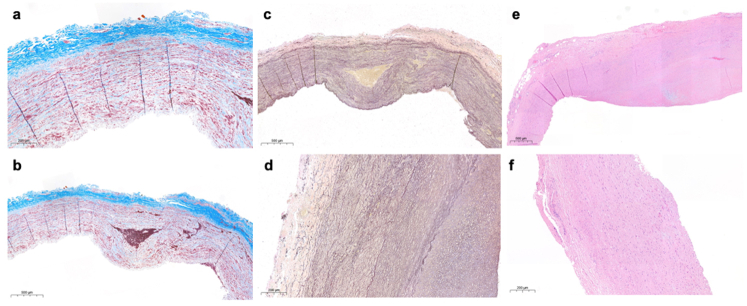
The histological examination of the intraoperative specimen (thoracic aneurysm) reveals a moderate-grade non-inflammatory degenerative aortic-iliac disease with severe lysis of the elastic fibers of the tunica media of the aorta.

Postoperative computed tomography angiography showed no complications after TAAA reconstruction (Supplementary Fig 2, online only). The postoperative course was uneventful and the patient was discharged on postoperative day 11. Histological examination (Supplementary Fig 3, online only) revealed a moderate-grade noninflammatory degenerative disease with severe lysis of the elastic fibers of the tunica media. A new genetic test panel was performed after the operation, at Yale University, using high-throughput sequencing to evaluate the genes associated with aortic aneurysm. The panel revealed two genetic variants of uncertain significance: the PKD1 gene and FBN1 gene. The patient gave his consent for publication.

## Discussion

We report a rare case of MAA, involving the supra-aortic trunks, as well as the thoracic, abdominal aorta, and renal arteries. Synchronous/metachronous aneurysms involving the thoracoabdominal aorta may be under-reported, especially when AAA is the first diagnosis,^11^ unlike synchronous peripheral aneurysms, which are commonly screened for and, thus, treated.^2^^,^^12^ A limited number of studies have described an association between AAA and TAA, and there are no established guidelines.^2^^,^^12^^,^^13^

Several etiologies of MAA have been proposed, including inflammation, infection, and HTADs. Multiple genetic syndromes are associated with MAA, and the most prevalent conditions include Marfan syndrome, Ehlers-Danlos syndrome, Loeys-Dietz syndrome, familial thoracic aortic aneurysms and dissections, and autosomal-dominant polycystic kidney disease.^14^^,^^15^

This article presents a compelling case illustrating the challenges in the decision-making process and treatment of MAA and the successful treatment of the condition using OSR. Despite the first genetic panel, the suspicion for HTAD was high, owing primarily to the patient's age and the absence of atherosclerotic signs.

The primary concern was related to the juxtarenal AAA, which was very large and at very high risk of rupture. An endovascular approach was considered but not chosen because of the patient's young age and fitness, possible HTAD etiology, and anatomical limitation to the use of off-the-shelf devices. OSR of the TAAA could have been accomplished in a single operation; however, from the left thoracophrenolaparotomy, the distal right renal artery cannot be reached. Moreover, staging of the procedure could mitigate the risk of spinal cord ischemia (SCI).

The second concern in the decision-making process regards the choice of a single-stage or two-stage OSR. Simultaneous treatment has been associated with severe perioperative complications. At the same time, sequential repair necessitates two major surgical procedures, with the risk of rupture of the residual lesion while waiting. Regardless of the approach timing and type, these patients require extensive aortic treatment and face a high risk of perioperative complications (eg, SCI).

During TAAA OSR, spinal cord protection relies primarily on cerebrospinal fluid drainage and neuromonitoring with motor evoked potentials and somatosensory evoked potentials. Cerebrospinal fluid drainage was implemented for routine use in extents I, II, and III at our institution in 2003 and upgraded to the automated LiquoGuard system in 2013.^10^^,^^16^

In MAA, Etz et al^17^ showed that SCI may occur less frequently when the repair is performed in a two-stage approach rather than a single procedure. Some authors advocate the increased rate of complications if two major surgeries are performed in two different steps, including the risk of rupture of the residual lesion in the interval time between the operations.^17^^,^^18^

Long-term results have not been widely reported in MAA. In their experience, Crawford and Cohen^19^ reported that long-term survival was better in patients with complete aortic treatment because most of the early deaths in the sequential intervention were caused by rupture of the second aneurysm. In contrast, Gloviczki et al^20^ showed improved survival at 3 and 5 years after sequential repair, although it was not statistically significant. Scali et al^21^ demonstrated an overall survival of 81% at 1 year for simultaneous totally endovascular repair.

## Conclusions

Although OSR is technically challenging and potentially associated with significant perioperative morbidity and mortality, in this young patient with MAA and possible heritable etiology showed to be effective, performed without any complication in a high-volume aortic center. The timing and staging strategy for the management is still a major issue. Given the need for multidisciplinary management, patients should be referred to high-volume aortic centers with a special interest in rarer conditions such as inflammatory, infected, and HTAD aneurysms.

## Funding

None.

## Disclosures

None.
